# Taiwan’s Strategy Toward Measles Elimination

**DOI:** 10.3390/vaccines14040361

**Published:** 2026-04-17

**Authors:** Fu-Tien Lin, Chin-Hui Yang, Wen-Yueh Cheng, Jean-Yun Chang

**Affiliations:** 1 Division of Acute Infectious Diseases, Taiwan Centers for Disease Control, Ministry of Health and Welfare, No. 6 Linsen S. Road, Taipei City 10050, Taiwan; stephen@cdc.gov.tw (F.-T.L.);; 2 Center for Diagnostics and Vaccine Development, Taiwan Centers for Disease Control, Ministry of Health and Welfare, No. 128, Sec. 2, Academia Rd., Nangang Dist., Taipei City 115201, Taiwan; dianechengwy@gmail.com

**Keywords:** measles elimination, MMR vaccine, seroprevalence, waning immunity, immunization program, genotypes, Taiwan

## Abstract

Background: Sustaining measles elimination in the post-elimination era presents increasing challenges due to global resurgence and waning vaccine-induced immunity. We aimed to evaluate epidemiological trends, vaccination strategies, and population immunity associated with achieving and maintaining measles elimination in Taiwan. Methods: We conducted a comprehensive analysis of national surveillance data from 1991 to 2024, including case notifications, viral genotypes, vaccination coverage rates, and surveillance performance indicators. Three population-based seroprevalence surveys conducted between 2002 and 2020 were reviewed to assess age-specific immunity. Descriptive analyses were performed to characterize long-term epidemiological and immunological trends. Results: From 1993 to 2024, the annual number of measles cases remained consistently below 50, except in 2019. Vaccination coverage for both MMR1 and MMR2 has exceeded 95% since 1998, with MMR1 coverage remaining above 97% between 2009 and 2024. Genotyping evidence confirms the interruption of endemic transmission since 2006; furthermore, as of 2024, no continuous chains of transmission lasting longer than 12 months have been recorded. National seroprevalence surveys monitoring measles-specific IgG antibodies revealed declining antibody levels among adolescents and young adults, with seropositivity as low as 36.7% in specific cohorts. Despite this, transmission following importations has remained limited, with minimal secondary spread. Conclusions: Taiwan has successfully sustained measles elimination through high vaccination coverage, robust surveillance, and targeted interventions. Although serological evidence indicates waning immunity, epidemiological data suggest preserved population-level protection, likely mediated by immunological memory. Targeted booster strategies for high-risk groups may be more appropriate than universal additional dosing in post-elimination settings.

## 1. Introduction

Measles remains one of the most contagious vaccine-preventable diseases despite the availability of a safe and effective vaccine. Although global vaccination efforts have substantially reduced morbidity and mortality, recent resurgences highlight the fragility of measles elimination, particularly amid declining routine immunization coverage and increased international travel [1]. Prior to the introduction of the measles vaccine in 1963 and subsequent widespread immunization efforts, major epidemics occurred approximately every two to three years, resulting in an estimated 2.6 million deaths annually [2]. Nearly all children acquired measles by age 15 years [3]. During 2000–2023, an estimated 60.3 million measles deaths were averted by vaccination [4].

Taiwan introduced a live-attenuated measles vaccine (MV) in 1968 and, in 1978, implemented a routine immunization program providing MV to infants at 9 and 15 months of age. However, suboptimal early coverage led to periodic outbreaks, with three major outbreaks in 1985 (n = 2219), 1988 (n = 1386), and 1989 (n = 1060). In response, the Department of Health (reorganized as the Ministry of Health and Welfare in 2013) launched a comprehensive national program from 1991 to 1996 to eradicate poliomyelitis, measles, congenital rubella syndrome (CRS), and neonatal tetanus (NT) [5]. In the ensuing years, the immunization and surveillance programs were strengthened and fully integrated into the healthcare system. Under the program, measures were implemented to strengthen surveillance systems, case investigation, vaccination coverage, vaccine cold-chain management, physician and healthcare worker education, and diagnostic technology. Measles had been effectively controlled. In 2010, the national measles elimination program was reformed to align with the World Health Organization (WHO) Western Pacific Regional Office (WPRO) guidelines, which set a regional target for measles elimination [6,7].

This study provides a comprehensive evaluation of Taiwan’s strategy for measles elimination, drawing on over three decades of integrated epidemiological, immunological, and virological data. These strategies, implemented in Taiwan, have been the primary drivers of achieving and sustaining measles elimination, in alignment with WHO guidelines [7]. In addition, we examine the implications of waning vaccine-induced immunity in a post-elimination setting.

## 2. Materials and Methods

### 2.1. Surveillance and Immunization Data Collection

Measles is a notifiable disease in Taiwan, requiring reporting within 24 h and laboratory confirmation. Epidemiological and laboratory data were obtained from the Taiwan Centers for Disease Control (Taiwan CDC). All specimens for reporting suspected cases were sent to the Center for Diagnostics and Vaccine Development of the Taiwan CDC for Disease Control for laboratory testing. Vaccination records were retrieved from the National Immunization Information System (NIIS), a centralized database covering childhood immunizations nationwide.

### 2.2. Data Analysis and Serological Test

To evaluate epidemiological trends and vaccination coverage, we analyzed national measles surveillance and immunization records from 1991 to 2024. Data were derived from all notified cases maintained by the Communicable Diseases Division of Taiwan CDC and NIIS. Variables retrieved for each case included age, gender, occupation, symptom onset and diagnosis dates, clinical symptoms, and travel history. Laboratory test results were also integrated into the dataset.

Furthermore, to monitor immunity trends across age groups and establish a robust evidence base for policy refinement, three key seroprevalence surveys were conducted between 2002 and 2020. Serum measles-specific immunoglobulin G (IgG) antibodies were measured in all three seroprevalence surveys using commercially available enzyme-linked immunosorbent assay (ELISA) kits. Two assay systems were used: Enzygnost^®^ Anti-Measles Virus/IgG (Siemens, Munich, Germany) and Anti-Measles Virus ELISA (Euroimmun, Lübeck, Germany), according to the manufacturers’ instructions.

Serostatus was interpreted based on assay-specific cut-off values. For the Siemens Enzygnost^®^ assay, results were classified as positive (>300 mIU/mL) or equivocal (150–300 mIU/mL). For the Euroimmun assay, results were considered positive at ≥275 IU/L and equivocal at 200 to <275 IU/L.

- The first survey (2002–2007) utilized residual sera from a cohort study titled “Prevalence Survey of Triple-High Status” (participants aged ≥15 years) [8].
- The second survey (2007–2008) employed a multi-stratified design to sample the general population, ranging from infants (<1 year) to seniors (≥65 years) [9].
- The third survey (2019–2020) utilized residual blood samples from the “Taiwan National Immunity Longitudinal Survey” (participants aged 3–59 years) [10].

### 2.3. Statistical Analysis

For the 2019–2020 measles seroprevalence survey, participants were classified into three birth cohorts: (1) natural infection cohort (aged 45–59 years), (2) pre-campaign vaccination cohort (aged 25–44 years), and (3) post-campaign vaccination cohort (aged 3–24 years). Pearson’s chi-square test was used to examine the association between birth cohort and serostatus (seropositive vs. seronegative). In addition, binary logistic regression analysis was performed with seropositivity (1 = positive, 0 = negative) as the dependent variable and birth cohort as a categorical independent variable, using the pre-campaign vaccination cohort as the reference group. Odds ratios (ORs) and 95% confidence intervals (CIs) were estimated. A two-sided *p*-value < 0.05 was considered statistically significant.

## 3. Results

### 3.1. Long-Term Epidemiological Trends

**Figure 1:**
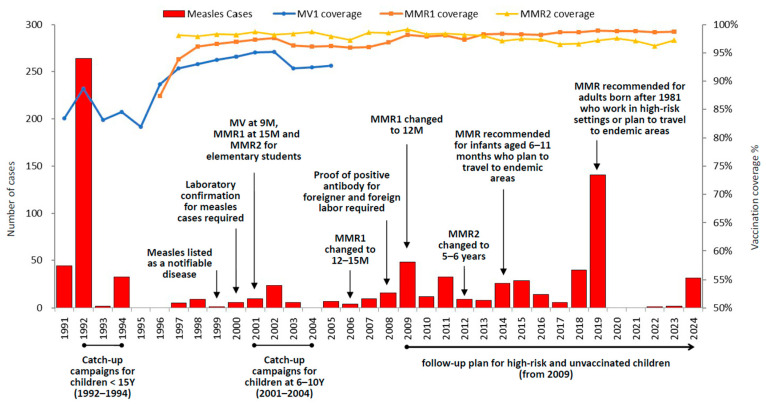
Trends of measles cases and the measles control program in Taiwan from 1991 to 2024.

From 1993 to 2024, measles incidence remained consistently low, and the annual number of measles cases remained below 50 [11], corresponding to an incidence rate of less than 2.1 cases per million population—except in 2019, when a resurgence of global measles activity led to multiple imported cases and subsequent clusters in healthcare settings and restaurants (Figure 1) [12]. Between 1993 and 2024, a total of 534 confirmed cases were reported over the 32-year period, including 342 (64%) non-imported cases and 192 (36%) imported cases. Most of the imported cases were either infected in Southeast Asian countries (53.7%) or China (27.5%). Prior to 2016, imported cases primarily originated from Mainland China. However, after 2016, the majority of infections shifted to Southeast Asian countries. Notably, no imported cases from China have been recorded since 2020.

**Figure 2:**
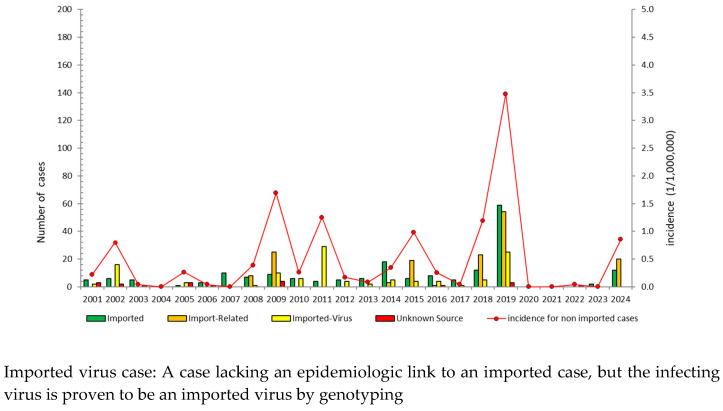
Trends in measles cases and the incidence of non-imported cases in Taiwan from 2001 to 2024. The average annual incidence rate for non-imported cases from 2001 to 2024 was 0.4 per 1 million, and all the annual incidence rates for non-imported cases were less than 1 per 1 million, except for 2009, 2011, 2018, and 2019.

**Figure 3:**
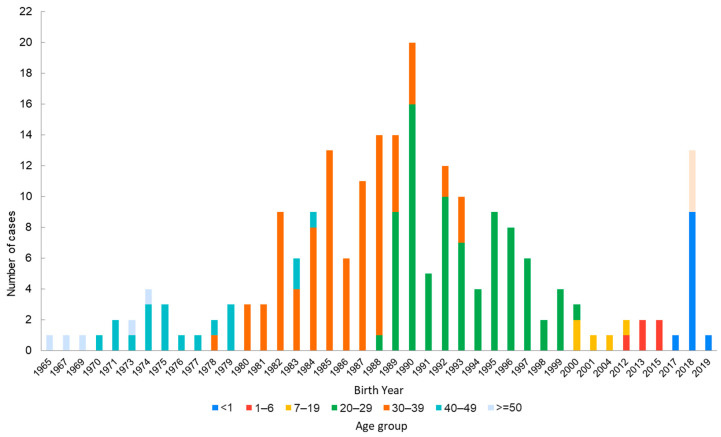
Distribution of measles cases by birth year and age from 2018 to 2024. Cases born in or before 1982 accounted for only 13% of all cases; furthermore, only one case was born before 1966, and this individual was an immunocompromised patient with cancer.

**Figure 4:**
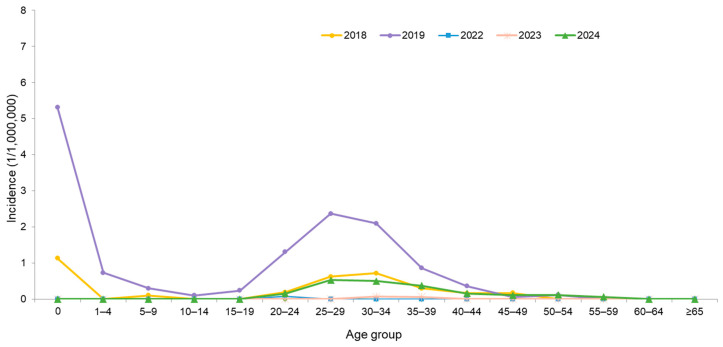
Age-specific Incidence of Measles Cases in Taiwan from 2018 to 2024 *. In 2019, the average single-year population of the 25–29 age group was 1.9 times that of the age 0 cohort. The elevated infant measles incidence that year is attributed to a diminished birth cohort combined with an increase in imported cases and subsequent household clusters linked to severe regional outbreaks in neighboring countries. * There were no confirmed cases in 2020 and 2021.

From 2001 to 2024, a total of 478 confirmed cases were reported, including 39.5% imported cases. Most domestic cases were epidemiologically linked to importations (Figure 2). Over the past seven years (2018–2024), there were 216 confirmed cases, including 131 (60.7%) non-imported cases and 85 (39.3%) imported cases. The top three countries of origin for imported cases have been Thailand (24 cases), Vietnam (23 cases), and the Philippines (9 cases). Most cases occurred among individuals born after 1981 (Figure 3). They were predominantly young adults aged 20–39, as well as infants under 1 year old who were too young to be vaccinated (Figure 3 and Figure 4).

### 3.2. Vaccination Policy and Coverage Rates

Taiwan has had a routine measles vaccination schedule for infants and children since 1978, with two doses of MV given at 9 months and 15 months. In 1991, the eradication program was initiated, and a regulation requiring the examination of immunization records for all elementary school entrants was established to monitor vaccination status and improve vaccination coverage rates. In 1992, one dose of MV was given at 9 months of age, and one dose of measles–mumps–rubella vaccine (MMR) was administered at 15 months of age. To improve coverage rates and ensure that people born after 1976 received at least 2 doses of measles-containing vaccine (MCV) by age 1, two catch-up campaigns were implemented. From 1992 to 1994, the campaign targeted junior high school students through preschool children (1976 to 1990 birth cohort), while the campaign from 2001 to 2004 was aimed at elementary school students (1990 to 1994 birth cohort). Since 2001, the second dose of the measles–mumps–rubella vaccine (MMR2) has been routinely administered to elementary school students. In 2006, the first dose of the measles–mumps–rubella vaccine (MMR1) was changed from 15 months to 12–15 months. However, there were some unvaccinated children aged 1 to 2 years old who were infected with measles and induced some clusters during 2008 and 2009. The time for administering MMR1 was revised from 12 to 15 months of age to 12 months of age since 2009. In 2012, the age for MMR2 was changed to 5 to 6 years old (Figure 1).

Between 1998 and 2024, immunization coverage for MMR1 by age 2 years was consistently high, at 95.9% or higher. Regarding MMR2, coverage rates ranged from 96.1% to 99.1% among primary school entrants (aged 6–7 years) from 1997 to 2011, and from 95.7% to 98.2% among children aged 5–6 years from 2012 to 2024. The coverage for routine MMR1 and MMR2 remained high at 98.8% and 97.4% as of 2024, respectively (Figure 1).

### 3.3. Quality of Epidemiological and Laboratory Surveillance Systems

Prior to 1999, measles was classified as a reportable rather than a notifiable disease in Taiwan. To enhance detection sensitivity, the Taiwan CDC implemented a dual active and passive surveillance framework. In 1991, a “zero-reporting system” was established, targeting clinical sites specialized in pediatrics, internal medicine, and family medicine. Local health authorities conducted weekly telephone inquiries to monitor suspected cases of acute flaccid paralysis, measles, rubella, and neonatal tetanus. These active surveillance data were cross-referenced with the passive Notifiable Disease Surveillance System. Following a period of stable epidemiological control, the zero-reporting measure was discontinued in 2015.

In 2007, Taiwan CDC began using the Laboratory Surveillance System—originally designed for influenza and enterovirus—to screen patients with symptoms such as rash, fever, cough, coryza, or conjunctivitis. Between 2007 and 2024, this proactive system identified only 2 confirmed measles cases, reflecting low community transmission. Taiwan has a high-quality surveillance system that is sensitive enough to detect imported and import-related cases.

Since 2000, laboratory confirmation has been mandatory for all suspected measles cases. Standardized specimen collection (throat swabs, urine, and whole blood) for virologic testing was formalized in 2002. During the third phase of the measles eradication program (2002–2006), there were significant improvements in the rates of adequate specimen collection and epidemiological investigation (Figure 1). To maintain high surveillance quality, the Taiwan CDC established performance indicators to assess local health departments’ performance. In accordance with the WHO Western Pacific Regional Office (WPRO) guidelines for monitoring measles elimination [13], surveillance quality indicators from 2020 to 2024 were evaluated. Although the annual reporting rates of discarded non-measles cases fell below the target threshold between 2020 and 2023—a decline primarily attributed to reduced clinical suspicion and reporting activity following two years of zero cases and subsequent minimal incidence (1–2 cases)—all other surveillance performance indicators consistently exceeded established targets (Table 1).

### 3.4. Seroprevalence Survey

The measles seroprevalence surveys conducted between 2002 and 2007 revealed that natural infection cohorts born before 1976 maintained high seropositivity rates (93.3–98.5%). The 1976–1986 birth cohorts had a lower measles seropositive rate (81.7–84.7%) than that of the 1969–1975 cohorts (93.3–94.0%) (Table 2A). These findings suggest that vaccine-induced immunity may last for a shorter period than that acquired through natural infection. Longitudinal observation of the 1976–1986 cohort showed a 3% decline in seropositivity rate over a five-year period. Notably, the 2007–2008 study found that the seropositivity rates in the 1982–1987 cohort dropped to 50.6%, while the equivocal rate rose to 30.6% (Table 2B). The declining seropositive rate in vaccinated cohorts might be due to a lack of natural booster infections and waning immunity [9].

A recent seroprevalence survey conducted between 2019 and 2020 found an overall measles seropositivity rate of approximately 70% (1683/2400). Age-specific analysis revealed a U-shaped distribution of antibody prevalence. Seropositivity rates for ages 10–39 years were consistently below 80%, with the nadir in the 15–19 age group (36.7%). Conversely, the highest seropositivity was identified in the 55–59 age group (97.4%), followed by the 3–4 and 50–54 age groups (both at 93%) (Table 2B).

**Figure 5:**
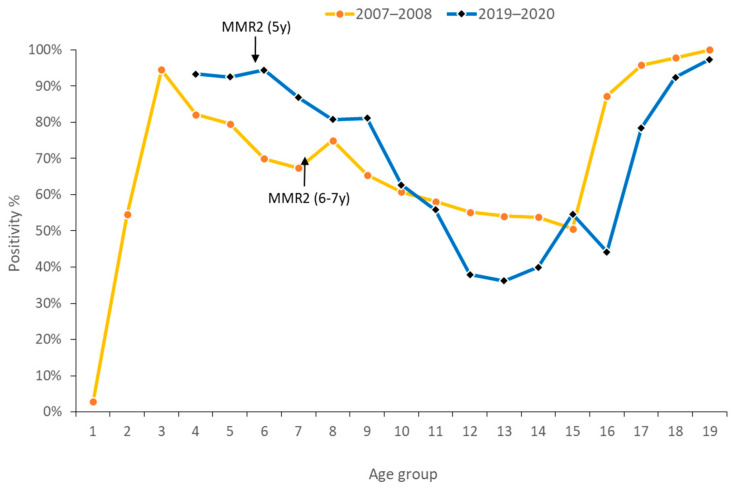
Comparison of seroprevalence of measles by age conducted in 2007–2018 and 2019–2020. Following the 2012 policy shift, the MMR2 vaccination schedule was advanced from primary school entry (ages 6–7 years) to age 5 years. Consequently, the MMR2 vaccination age was 6–7 years during the 2007–2008 survey period, whereas it was 5 years during the 2019–2020 survey.

To evaluate whether the birth-year threshold (born before 1981) for presumed measles immunity required revision, the Taiwan CDC conducted a second national seroprevalence survey in 2019–2020, 12 years after the previous assessment. Age-specific analysis across both surveys revealed a consistent U-shaped distribution of antibody prevalence (Figure 5). Specifically, seropositivity peaked at approximately 2 years of age (the first MMR dose is administered at 12–15 months), followed by a gradual decline. While a transient increase in seropositivity was observed following the second MMR dose (administered at age 5 or 6–7), it subsequently declined with age. In the 2007–2008 and 2019–2020 surveys, antibody positivity increased significantly among individuals aged 26 and 36 years, respectively. These cohorts, representing the pre-1981 birth generations, exhibited markedly higher seropositivity than those born in the vaccination era; in both surveys, seropositivity exceeded 97% for individuals aged 55 and older (Table 2B). However, the 2019–2020 survey identified a more pronounced decline in the adolescent population (aged 11–18) compared to the same age group in the 2007–2008 study. Notably, seropositivity reached a nadir of only 36% in the 17–18 age group, suggesting that vaccine-induced antibodies drop below 40% approximately ten years after the MMR2.

### 3.5. Genotyping Evidence

Genotyping of isolated measles viruses in Taiwan from 2001 to 2024 is summarized in Table 3. Between 2001 and 2017, genotype H1 was the most prevalent, identified in 56.1% (120/214) of all detected viruses. Although H1 was detected annually (except in 2004 and 2011), 88.7% (86/97) of genotype H1 cases identified from 2006 to 2017 were linked to known imported cases. During this period, 11 cases (one in 2008, three in 2012, four in 2015, and three in 2016) had no traceable source. Their viral sequences were closely related to lineages circulating endemically in China [14,15,16], indicating that genotype H1 was not an endemic genotype in Taiwan after 2006. Other genotypes, including D3, D4, D5, D8, D9, G3, and B3, were detected sporadically and were primarily associated with imported cases, with no evidence of sustained annual transmission [16].

Since 2018, measles virus genotypes B3 and D8 have been the only strains circulating in Taiwan. In 2018, 35 cases of genotype D8 were confirmed, 31 of which were import-related, while three cases were identified as genotype B3 (including two import-related cases). Although the 2019 epidemic was characterized by multiple importations and subsequent secondary transmission, strict border controls during the COVID-19 pandemic (2020–2021) resulted in zero confirmed cases, indicating no endemic transmission. Following this period, two imported D8 cases were reported in 2023. In 2024, 20 cases of genotype B3 were confirmed (18 of which were import-related), alongside 12 cases of genotype D8. Of the D8 cases, seven were import-related, and five formed a local cluster. Sequence analysis of the five local cluster cases revealed that they differed from the D8 sequences of the 2023 cases and were identical to those of cases imported from Thailand and Cambodia during the same period.

### 3.6. Risk Assessment and Continuous Prevention and Control Interventions

Taiwan CDC has established a multi-year, mid-to-long-term plan that includes measures for the prevention and control of measles. The plan aims to increase the MMR vaccination rate to achieve the elimination of measles. It ensures that children of all age groups receive appropriate vaccinations. Taiwan CDC has established performance assessment indicators for local health departments to achieve and maintain the goal of measles elimination. These indicators include the MMR1 vaccine coverage rate, with targets of ≥95% national coverage and ≥ 90% coverage in all townships, counties, and cities.

In order to prevent the importation of measles virus into Taiwan, Taiwan CDC drew up a guideline to strengthen the implementation of necessary quarantine practices in 2007. Since 2009, all foreigners applying for a resident or settlement visa are required to submit a positive measles/rubella antibody report or immunization certificate. In addition, the mandatory quarantine inspection and examination for foreign laborers prior to entry also include the submission of a positive measles/rubella antibody report or an immunization certificate.

In Taiwan, a significant proportion of foreign spouses originate from China and Southeast Asia. Frequent cross-border travel by these families increases the risk of measles virus exposure among unvaccinated children. During 2008–2009, severe measles outbreaks were reported in several neighboring countries, including China, Cambodia, Malaysia, the Philippines, and Vietnam. Following these regional surges, Taiwan recorded 16 imported cases during this period, primarily from China (n = 7) and Vietnam (n = 6). Notably, six of these cases involved children aged 0–4 years born to foreign spouses. These infections occurred either in infants below the eligible vaccination age or in children who traveled to high-endemicity regions prior to receiving their scheduled MCV. Upon returning to Taiwan, one infant (<1 year) and two toddlers (1-year-old) subsequently initiated hospital-based clusters. To mitigate transmission risks in children aged 1–6 years, the administration age for the MMR1 was standardized to 12 months in April 2009, narrowing the previous 12–15-month window. Furthermore, the Taiwan CDC integrated the NIIS with National Immigration Agency records to enhance the surveillance of unvaccinated children. Under this integrated framework, the NIIS automatically alerts local health agencies when a child without a documented MMR record arrives in Taiwan. Public health nurses then conduct active follow-up and vaccination outreach. Through these strategic applications of the NIIS [17], MMR1 coverage has consistently exceeded 97% since 2009 (Figure 1), resulting in a significant decline in the annual measles case count among the 1–6 age cohort (Table 4).

In 2014, Taiwan Advisory Committee on Immunization Practices (ACIP) recommended that infants aged 6–11 months, as well as individuals born after 1981, receive one dose of the MMR vaccine prior to travel to high-risk areas. Local health centers were also instructed to offer MMR vaccination to infants aged 6–11 months, preparing for international travel.

Due to ongoing measles outbreaks in healthcare settings and reported transmission involving healthcare personnel, it is recommended that all healthcare personnel who do not meet any of the criteria for presumptive evidence of measles immunity to receive one dose of MMR vaccine. Starting from 2023, the Hospital Infection Control Audit Standards began requiring hospitals to establish comprehensive vaccination plans according to relevant guidelines. These plans include ensuring that high-risk departments and all new hospital staff provide presumptive evidence of measles immunity.

## 4. Discussion

Taiwan introduced the measles vaccine into its routine childhood immunization program in 1978. In the early stages, suboptimal vaccination coverage led to periodic outbreaks every 2 to 3 years. However, measles was brought under effective control by 1993—only 15 years after the program’s inception. This milestone was primarily attributed to the 1991 implementation of the “Four-Phase Plan to Eradicate Poliomyelitis, Measles, Congenital Rubella Syndrome (CRS), and Neonatal Tetanus (NT).” Through this meticulously designed framework, Taiwan consistently developed and executed robust prevention and control strategies.

A pivotal policy established in 1991 mandated immunization record verification for all elementary school entrants. By implementing systematic reviews of routine vaccination histories and catch-up mechanisms for kindergarten and primary school students, Taiwan has maintained consistently high coverage rates for measles and other essential childhood vaccines. Furthermore, two important MMR vaccination catch-up campaigns targeting school-aged children were conducted between 1992 and 1994 and 2001–2004. These initiatives significantly increased measles vaccination rates and national herd immunity. Given that school enrollment in Taiwan exceeds 99.9%, school-based mass vaccination campaigns have proven highly effective. For instance, the MMR vaccination campaign conducted from 2001 to 2004, which targeted students in the 1st through 5th grades, achieved a coverage rate exceeding 98% for each grade level. Both MMR1 and MMR2 coverage rates have remained above 95% since 1998. By utilizing these two targeted campaigns, the eradication program enabled birth cohorts from 1976 to 1994 to rapidly achieve high vaccination rates and comprehensive immunity. This strategic approach successfully closed the immunity gaps present in earlier cohorts. Consequently, from 1995 to 2008, annual incidence rates for non-imported cases remained below 1 per million.

Genotypic analysis from 2001 to 2024 further corroborates these findings (Table 3). Genotype H1 cases, mostly originating from China, were detected annually between 2001 and 2017 (excluding 2004 and 2011). Epidemiological evidence since 2006 indicates that the majority of genotype H1 cases were import-related. Following China’s intensified elimination efforts, its national incidence has declined sharply since 2017. Correspondingly, genotype H1 has not been detected in Taiwan since 2018, having been replaced by genotypes D8 and B3 from Southeast Asia. This genotyping evidence confirms the interruption of endemic transmission after 2006. According to the WHO verification criteria [4,7,18], Taiwan has successfully sustained measles elimination.

The global resurgence of measles during 2018–2019 led to a significant increase in imported cases in Taiwan. In 2018, an imported index case triggered a notable cluster involving airline crew members, passengers, and ground staff. This was followed by multiple importations in 2019, resulting in several healthcare-associated clusters. Crucially, epidemiological investigations revealed that many infected healthcare workers had documented two-dose MMR vaccination histories. These occurrences suggest that infection may occur despite prior vaccination, potentially due to high-dose exposure in clinical settings or waning vaccine-induced immunity.

While these outbreaks were primarily contained within institutional settings and limited to identified contacts, they prompted a critical re-evaluation of national policy. Drawing on domestic and international evidence on measles antibody decline associated with MMR2 response [9,19], the Taiwan Advisory Committee on Immunization Practices (ACIP) established revised operational criteria for measles and rubella immunity in 2019. Under these guidelines, an individual is considered immune if they meet at least one of the following criteria:

- Birth year prior to 1981 (provided the individual is not immunocompromised);
- Laboratory-confirmed history of measles or rubella infection;
- Serological evidence of positive measles/rubella antibody titers within the previous five years;
- Documented administration of two MMR doses (with a minimum interval of 28 days) after one year of age, provided the final dose was received within the last 15 years.

To safeguard healthcare infrastructure, the Taiwan CDC integrated the MMR vaccination status of healthcare workers born in or after 1981 into national hospital infection control audit standards. Hospitals were mandated to implement comprehensive catch-up programs, providing an additional MMR dose to healthcare workers who either lacked a two-dose history or had received their last dose more than 15 years prior. Furthermore, based on risk assessments derived from the 2018–2019 clusters, the Taiwan CDC identified high-risk groups for infection and transmission, including travelers to endemic areas, healthcare workers, childcare workers, and personnel with frequent professional contact with foreign nationals. A booster MMR dose is formally recommended for individuals in these high-risk categories born in or after 1981 who do not meet the immunity criteria.

While Taiwan encountered multiple measles clusters in 2019 and a significant hospital-associated outbreak in 2024, transmission was effectively contained. During the COVID-19 pandemic, stringent border controls led to zero confirmed cases from 2020 to 2021, further demonstrating the absence of endemic transmission. Notably, the 2024 hospital cluster was confined to staff and patients, with no evidence of community spread. Since 2006, continuous surveillance and rigorous epidemiological investigations have confirmed that no transmission chains persisted beyond 12 months, substantiating Taiwan’s success in sustaining its measles elimination status.

Synthesizing these lines of evidence, Taiwan achieved effective control and elimination shortly after the vaccine’s introduction, significantly earlier than many neighboring countries. However, this success has led to a lack of “natural boosting” from the circulating wild-type virus. The 2007–2008 seroprevalence survey revealed that antibody levels waned over time in post-1981 vaccination era cohorts. The seropositivity rate reached its nadir in the 21–25 age group—approximately 15 years after MMR2—at only 50%, with an additional 30.6% falling into the equivocal range. In contrast, the naturally infected cohorts born before 1972 maintained seropositivity rates above 95%. For those born between 1972 and 1982, the seropositivity rate remained near 90% (Table 2B), likely because they were either infected naturally during the early stages of the vaccination program or received natural boosting from lingering outbreaks. These findings align with studies from other highly immunized or eliminated countries, showing significantly higher seroprevalence in older, naturally infected populations and notable waning immunity in young adults born after the implementation of national vaccination programs [9,19,20,21,22].

For the 2019–2020 seroprevalence survey, we performed supplementary statistical analyses stratified by birth cohort. Participants were categorized into three distinct groups based on their historical exposure to the virus and vaccination programs:

1. Natural infection cohort: Aged 45–59 years (born before 1976);
2. Pre-campaign vaccination cohort: Aged 25–44 years (born 1976–1991);
3. Post-campaign vaccination cohort: Aged 3–24 years (born after 1991).

A Pearson’s chi-square test was used to assess the association between birth cohort and serostatus (positive vs. negative). The results demonstrated a significant association (χ^2^ = 205.81, *p* < 0.001), indicating that seropositivity differed across cohorts. Furthermore, binary logistic regression analysis was performed with seropositivity (1 = positive, 0 = negative) as the dependent variable and birth cohort as a categorical independent variable, using the pre-campaign vaccination cohort as the reference group. The analysis showed that individuals in the natural infection cohort had significantly higher odds of seropositivity (OR = 8.97, 95% CI: 6.36–12.65), whereas those in the post-campaign vaccination cohort had only slightly higher odds (OR = 1.24, 95% CI: 1.02–1.50). To further examine heterogeneity within the post-campaign vaccination cohort, a Pearson’s chi-square test (5 × 2 contingency table) was conducted across five age groups, revealing a significant difference in seropositivity (χ^2^ = 165.3, *p* < 0.001). In addition, logistic regression analysis using the 15–19-year age group as the reference demonstrated that individuals aged 20–24, 10–14, 5–9, and 3–4 years had significantly higher odds of seropositivity (OR = 2.07, 3.16, 9.09, and 22.45, respectively; all *p* < 0.001). Within the post-campaign vaccination cohort, the 15–19 age group exhibited the lowest seropositivity rate, whereas a significantly higher rate was observed among those aged 20–24. This non-linear age trend suggests that measles IgG titers are influenced not only by the time elapsed since primary vaccination but also by targeted policy interventions. The elevated seropositivity in the 20–24 age group may be attributed to the 2019 supplemental immunization initiatives directed at high-risk individuals born after 1981. By encouraging catch-up MMR vaccinations among young adults, these measures likely enhanced vaccine coverage and population immunity within this specific bracket. Such policy-driven interventions may partially explain why the odds of seropositivity were higher in the 20–24 age group compared to the 15–19 age group.

The existing literature indicates that declining antibody titers do not necessarily indicate a total loss of protection. While vaccine-induced antibodies may wane or become undetectable, robust immunological memory often persists [23,24]. Most vaccinated individuals can mount a protective immune response upon exposure to the measles virus. Although secondary vaccine failure (SVF) may occur occasionally due to waning immunity, it does not appear to play a significant role in sustained measles transmission [23]. Despite the 2007–2008 findings showing a drop in seropositivity among young adults, Taiwan’s long-term epidemiological trends demonstrate that, since 1993, annual cases have remained extremely low and limited to cases imported from abroad (Figure 1). The absence of sustained community transmission suggests that the vaccinated cohorts retain enough immunological memory to maintain effective population-level immunity.

Conversely, the 21–25 age group in the 2019–2020 survey did not follow the expected downward trend but instead showed an increase in seropositivity (Figure 5). This deviation is likely attributable to the Taiwan CDC’s 2019 initiative, which recommended MMR booster vaccinations for adults born after 1981 who work in high-risk settings or plan travel to endemic areas. Furthermore, we observed that seropositivity among naturally infected individuals and vaccinated cohorts born before 1981 was lower in the 2019–2020 survey than in 2007–2008. This decline in both the “naturally infected” and “naturally boosted” cohorts suggests that antibody levels still wane over time in the absence of circulating wild-type virus, albeit at a slower rate than vaccine-induced immunity. Synthesizing epidemiological data from 2018 to 2024 (Figure 3) and evidence from the 2019–2020 survey, the Taiwan ACIP updated the criteria for measles immunity in 2025. The threshold of “Birth before 1981 (and not immunocompromised)” was formally adjusted to “Birth before 1966 (and not immunocompromised)”.

While our 2019–2020 survey indicated an overall measles IgG seroprevalence of only 71% among individuals aged 3–65 years—with a notable waning of immunity in adolescents and young adults—these figures must be interpreted with caution. A primary consideration is the inherent limitation of enzyme-linked immunosorbent assays (ELISA) compared to the plaque reduction neutralization test (PRNT). Although ELISA was employed in both the 2007–2008 and 2019–2020 surveys, PRNT remains the gold standard for assessing measles immunity, as it directly measures the functional neutralizing capacity of antibodies. Previous studies have demonstrated that individuals testing negative or equivocal by ELISA often possess protective neutralizing antibodies when re-evaluated via PRNT, suggesting that ELISA-based surveys frequently underestimate true population-level protection [23,24,25]. Furthermore, the discrepancy between the two surveys may be exacerbated by the use of different commercial kits. Modern assays exhibit varying sensitivities in detecting low-titer vaccine-induced immunity, which wanes over time, compared with the robust titers following natural infection. Specifically, diagnostic performance near the cut-off (equivocal) range is inconsistent across brands [26]. While the 2007–2008 survey utilized the Siemens Enzygnost kit, the 2019–2020 study transitioned to the Euroimmun kit to minimize false-positive rates. However, this shift may have inadvertently led to an overestimation of the “equivocal” or “Negative” proportion. Furthermore, analysis of the age distribution of confirmed measles cases from 2023 to 2024 revealed that most cases occurred in the age group born after 1981 (the 30–41 age group accounted for 70%). Therefore, it can be inferred that the actual proportion of measles antibodies in different age groups in Taiwan is not as low as indicated by this study’s results. Future research directions could focus on conducting paired ELISA-PRNT analyses within vaccinated cohorts experiencing antibody waning. Such studies would serve to verify that adolescents and young adults who test negative or equivocal by ELISA often still possess protective neutralizing antibodies, thereby confirming that population immunity in Taiwan remains sufficient.

This study faces certain limitations regarding the comparative analysis of measles seropositivity trends across age groups between the 2007–2008 and 2019–2020 surveys. These surveys were conducted by different contracted academic institutions using pre-defined age stratifications. Notably, the 2007–2008 data were obtained as secondary data from previously published literature. In the absence of raw participant records from these third-party institutions, it was not feasible to re-categorize or align the age cohorts into identical intervals for a direct longitudinal comparison. Furthermore, the use of different commercial diagnostic kits (reagents) across the two periods introduces potential variations in assay sensitivity and specificity, which may impact the direct comparability of seropositivity rates.

## 5. Conclusions

Monitoring of measles virus genotypes indicates the interruption of endemic measles transmission in Taiwan after 2006. According to WHO’s five lines of evidence for verification of measles elimination, Taiwan has successfully maintained measles elimination since 2006. Although serological waning is observed, the lack of large-scale outbreaks suggests that immunological memory provides sufficient population-level protection. Consequently, two documented MCV doses are considered adequate for immunity [27], and a universal third MMR dose remains unnecessary—especially as secondary vaccination failure (SVF) carries an exceedingly low transmission risk. Furthermore, evidence from hospital cluster investigations and a systematic review of individuals with secondary vaccination failure (SVF) indicates that the secondary attack rate from breakthrough infections is exceedingly low [28]. In response to the post-COVID-19 global resurgence, future efforts will transition from universal strategies to precision prevention. Based on 2019–2020 seroprevalence data, targeted boosters should prioritize high-risk groups, specifically healthcare workers who are more than 15 years post-vaccination. Ongoing longitudinal seroprevalence monitoring will be essential to ensure sustained vigilance against imported cases.

## Figures and Tables

**Table 1:** Performance of indicators to monitor progress toward measles elimination from 2020 to 2024 in Taiwan.

Indicators	Parameters	
	Target	2020–2024
**Incidence**		
Incidence of all measles cases per million total population (exclude imported cases)	<1	0.00–0.85
**Population immunity**		
National MCV1 coverage	≥95%	98.6–98.8%
National MCV2 coverage	≥95%	96.2–97.6%
**Quality of the surveillance system**		
**Epidemiologic surveillance indicators**		
% of suspected cases reported within 24 h	≥80%	96.9–100.0%
Annual reporting rate of discarded non-measles cases per 100,000 population at the national level *	≥2 per 100,000	0.39–2.35
% of suspected cases with adequate investigation	≥80%	100%
**Laboratory indicators**		
% of suspected cases with adequate blood specimen	≥80%	96.7–100%
% of blood specimens received at the TCDC laboratory within five days of collection	≥80%	100%
% of specimens with results reported within four days of receipt at the designated laboratory	≥80%	99.0–100%
% of outbreaks with specimens collected for virus detection	≥80%	100%
% of virus detection and genotyping completed within two months of receipt at the lab	≥80%	100%

Abbreviations: MCV, measles-containing vaccines; TCDC, Taiwan Centers for Disease Control. * The specimens obtained from reported measles and rubella cases undergo testing for both diseases as part of routine laboratory surveillance. Therefore, the numbers of suspected cases were combined for calculation.

**Table 2:** (A) Measles seroprevalence by birth cohort in Taiwan, 2002 and 2007. (B) Measles seroprevalence by age group in Taiwan, 2007–2008 and 2019–2020.

**(A)**						
Survey Year	Population Source	Birth Cohort		n	Seropositive (%)	Equivocal (%)
2002	Triple-high survey ≥15 years old	1956–1968		204	98.0	1.0
		1969–1975		300	94.0	5.0
		1976–1986		300	84.7	10.3
2007	Triple-high survey ≥15 years old	1956–1968		204	98.5	1.0
		1969–1975		300	93.3	4.0
		1976–1986		300	81.7	10.0
**(B)**						
Survey Year	Population Source	Age Group	Birth Cohort	n	Seropositive (%)	Equivocal (%)
2007–2008	To evaluate protections against vaccine-preventable diseases study in the general population in Taiwan	≥65	~1942	251	98.8	1.2
		56–65	1942–52	250	100.0	0.0
		46–55	1952–62	267	97.8	1.5
		36–45	1962–72	263	95.8	3.0
		26–35	1972–82	245	87.3	8.2
		21–25	1982–87	255	50.6	30.6
		17–20	1987–91	408	53.9	23.3
		13–16	1991–95	302	56.6	19.2
		9–12	1995–99	421	63.2	22.1
		5–8	1999–2002	409	71.9	17.1
		3–4	2002–2004	210	84.8	11.9
		2	2004–2005	127	94.5	3.1
		1	2005–2006	99	54.5	2.0
		<1	2006~	35	2.9	0.0
2019–2020	National Immunity Survey	55–59	1959–65	190	97.4	1.6
		50–54	1964–70	201	93.0	2.0
		45–49	1969–75	210	89.1	1.4
		40–44	1974–80	211	81.0	5.2
		35–39	1979–84	203	66.5	9.4
		30–34	1984–89	197	48.2	10.2
		25–29	1989–95	201	41.8	11.4
		20–24	1994–2000	209	54.6	6.7
		15–19	1999–2005	210	36.7	8.1
		10–14	2004–10	215	64.7	8.4
		5–9	2009–14	213	84.0	5.2
		3–4	2014–17	140	92.9	0.7

Abbreviations: n, sample size. All antibody measurements were performed using ELISA. Seropositive and equivocal were defined based on ELISA cutoffs (see Section 2.2).

**Table 3:** Genotypes of measles virus in Taiwan from 2001 to 2024.

Year	Measles Virus Genotypes								
	H1	D3	D4	D5	D8	D9	G3	B3	All
2001	5(3) ^a^	0	0	0	0	0	0	0	5(3)
2002	14(3)	1(1)	0	1	0	0	0	0	16(4)
2003	1(1)	2(2)	0	1(1)	0	1(1)	0	0	5(5)
2004	0	0	0	0	0	0	0	0	0
2005	3	0	0	1(1)	0	0	0	0	4(1)
2006	3(3)	0	0	0	0	0	0	0	3(3)
2007	3(3)	0	0	3(3)	0	1(1)	0	0	7(7)
2008	8(7)	0	0	1(1)	0	2(2)	0	0	11(10)
2009	30(30)	0	0	0	1(1)	0	10	0	41(31)
2010	3(3)	0	0	0	0	7(1)	0	0	10(4)
2011	0	0	2(2)	0	0	27(1)	0	0	29(3)
2012	5(2)	0	0	0	3(2)	0	0	0	8(4)
2013	5(5)	0	0	0	2	0	0	0	7(5)
2014	5(5)	0	0	0	4(4)	1(1)	0	12(7)	22(17)
2015	27(23)	0	0	0	0	0	0	0	27(23)
2016	7(4)	0	0	0	6(5)	0	0	0	13(9)
2017	1(1)	0	0	0	4(4)	0	0	1	6(5)
2018	0	0	0	0	35(31)	0	0	3(2)	38(33)
2019	0	0	0	0	102(82)	0	0	34(27)	136(109)
2020	0	0	0	0	0	0	0	0	0
2021	0	0	0	0	0	0	0	0	0
2022	0	0	0	0	0	0	0	0	0
2023	0	0	0	0	2(2)	0	0	0	2(2)
2024	0	0	0	0	12(7)	0	0	20(18)	32(25)
Total	120(93)	3(3)	2(2)	7(6)	171(138)	39(7)	10	70(54)	422(303)

^a^: The numbers in parentheses were associated with imported cases supported by epidemiological evidence.

**Table 4:** Annual distribution of measles cases among children aged 1–6 years from 2009 to 2024. ^a^: Documented administration of one MMR dose; ^b^: Foreign child; ^c^: Foreign national father refused MMR vaccination for the child.

Age (Years)	2009	2010	2011	2012	2013	2014	2015	2016	2017	2018	2019	2020	2021	2022	2023	2024
1	7	2	1	1	0	4	1 ^b^	1 ^c^	1	0	3	0	0	0	0	0
2	5	0	0	0	0	1 ^a^	0	0	0	0	0	0	0	0	0	0
3	1	0	0	0	0	0	0	0	0	0	2(1 ^a^,1 ^b^)	0	0	0	0	0
4	1	0	0	0	0	0	0	0	0	0	0	0	0	0	0	0
5	2	0	0	0	0	0	0	0	0	1 ^a^	1 ^a^	0	0	0	0	0
6	1	0	1	0	0	0	0	0	0	0	1 ^b^	0	0	0	0	0
**Total cases**	17	2	2	1	0	5	1	1	1	1	7	0	0	0	0	0

## Data Availability

The data presented in this study are also available on request from the corresponding author.

## Abbreviations

The following abbreviations are used in this manuscript:

MV	Measles Vaccine
CRS	Congenital Rubella Syndrome
NT	Neonatal Tetanus
WHO	World Health Organization
WPRO	Western Pacific Regional Office
Taiwan CDC	Taiwan Centers for Disease Control
NIIS	National Immunization Information System
MMR	Measles–Mumps–Rubella Vaccine
MCV	Measles-Containing Vaccine
MMR1	The First Dose of The Measles–Mumps–Rubella Vaccine
MMR2	The Second Dose of The Measles–Mumps–Rubella Vaccine
SVF	Secondary vaccine failure
Taiwan ACIP	Taiwan Advisory Committee on Immunization Practices
ELISA	Enzyme-Linked Immunosorbent Assays
PRNT	Plaque Reduction Neutralization Test
